# Digital variance angiography in patients undergoing lower limb arterial recanalization: cost–effectiveness analysis within the English healthcare setting

**DOI:** 10.57264/cer-2023-0068

**Published:** 2024-03-22

**Authors:** Amir Ansaripour, Eoin Moloney, Michael Branagan-Harris, Lorenzo Patrone, Mehdi Javanbakht

**Affiliations:** 1 Optimax Access Ltd, Hofplein, Rotterdam, 3032AC, The Netherlands; 2 Optimax Access Ltd, Kenneth Dibben House, Enterprise Rd, Chilworth, Southampton Science Park, Southampton, SO16 7NS, UK; 3 Device Access Ltd, Market Access Consultancy, University of Southampton Science Park, Southampton, SO16 7NS, UK; 4 West London Vascular & Interventional Centre, London North West University Healthcare NHS Trust, Harrow, HA1 3UJ, UK

**Keywords:** contrast media reduction, digital subtraction angiography, digital variance angiography, economic evaluation, imaging, radiation reduction

## Abstract

**Aim:**

Digital variance angiography (DVA) is a recently developed image processing method capable of improving image quality compared with the traditionally used digital subtraction angiography (DSA), among patients undergoing lower limb x-ray angiography. This study aims to explore the potential cost–effectiveness of DVA from an English National Health Service perspective.

**Materials & methods:**

A two-part economic model, consisting of a decision tree and a Markov model, was developed to consider the costs and health outcomes associated with the use of DVA as part of current practice imaging, compared with x-ray angiography using standard DSA. The model explored the impact of DVA on the development of acute kidney injury (AKI), chronic kidney disease and radiation-induced cancer over a lifetime horizon. Both deterministic and probabilistic analyses were performed to assess the cost per quality-adjusted life-year (QALY).

**Results:**

Base-case results indicate that DVA results in cost savings of £309 per patient, with QALYs also improving (+0.025) over a lifetime. As shown in sensitivity analysis, a key driver of model results is the relative risk (RR) reduction of contrast-associated acute kidney injury associated with use of DVA. The intervention also decreases the risk of carcinoma over a lifetime. Scenario analyses show that cost savings range from £310 to £553, with QALY gains ranging from 0.048 to 0.109 per patient.

**Conclusion:**

The use of DVA could result in a decrease in costs and an increase in QALYs over a lifetime, compared with existing imaging practice. The potential for this technology to offer an economically viable alternative to existing image processing methods, through a reduction in contrast media volume and radiation exposure, has been demonstrated.

Digital variance angiography (DVA) is a recently developed image processing method, which shows potential to reduce the rate of clinical complications seen with current methods such as digital subtraction angiography (DSA), among patients undergoing lower limb x-ray angiography [1,2]. DSA may be used to visualize the vascular anatomy in selected patients [3] and is the current reference-standard image processing method for vessel visualization during interventional procedures [1]. The technique involves the injection of contrast medium into an artery or vein, while a physician acquires a series of images using x-ray angiography. Computer software is then used to subtract the image acquired immediately before contrast medium injection, from the images obtained post-contrast medium injection, to produce a final image set that allows the clear visualization of blood vessels without the disturbing anatomical background which includes soft tissue and bones [4].

The processing method is extensively used during endovascular procedures [5], with DSA being of particular benefit when undertaking examination prior to corrective surgical procedures involving arteries supplying the legs, brain and heart [4]. While advancements have been made in DSA technology over the past number of years, with progression from 2D to 3D and 4D imaging [6,7], drawbacks of the technique include significant radiation exposure, which has been demonstrated to exceed the one associated with DVA [1].

The innovative DVA technology is based on kinetic imaging [8], and allows for enhancement of contrast media (CM)-induced changes and suppression of background noise [1,9]; in this way, the overall image quality is improved [1,9–11]. Gyánó *et al.* demonstrated that use of DVA led to a significantly higher visual score than DSA, while allowing for an approximate 70% reduction in DSA-related radiation exposure, among patients undergoing lower limb x-ray angiography [1]. Similarly, Thomas *et al.* demonstrated that DVA significantly improved visual quality compared with DSA during lower limb interventions, irrespective of the CM agent used [9]. Bastian *et al.* also highlighted the superior image quality associated with DSA, when performing lower limb angiography with metal implants, which may be effectively used for dose management (radiation and CM reduction) [12].

While existing clinical results are promising, information on the cost–effectiveness of DVA is limited. This analysis describes an economic model developed to assess the cost–effectiveness of DVA during lower limb, or abdomen, x-ray angiography procedures when compared with existing imaging practice, i.e., DSA alone, from an English National Health Service (NHS) perspective.

## Materials & methods

An economic decision model was developed in Microsoft Excel to assess the cost–effectiveness of using DVA, as an add-on image processing technology in combination with current practice imaging (DSA), among a cohort of patients requiring lower limb, or abdomen, x-ray angiography. A decision tree followed by a Markov model was developed to reflect the treatment pathway, including the short-term clinical pathways of a patient undergoing lower limb, or abdomen, imaging involving DVA alongside current practice (intervention), or based on current practice imaging with DSA alone (comparator) (decision tree) and the long-term clinical pathways associated with the potential development of complications including contrast-associated acute kidney injury (CA-AKI) followed by chronic kidney disease (CKD), radiation-induced (RI) cancer and, ultimately, an increase in cardiovascular event (myocardial infarction [MI]) and mortality rates (Markov model).

The analysis was performed from an English NHS perspective over a lifetime horizon (one-year time cycles) to assess the long-term clinical and economic impact of the introduction of imaging using DVA technology. The Professional Society for Health Economics and Outcomes Research (ISPOR)'s ‘Principles of Good Practice for Decision Analytic Modeling in HealthCare Evaluation’ were followed in developing and formulating the economic analysis [13]. Costs and benefits were discounted at a rate of 3.5%, as recommended in the NICE methods guide for treatments that result in long-term health benefits [14].

### Model overview

**Figure 1.**
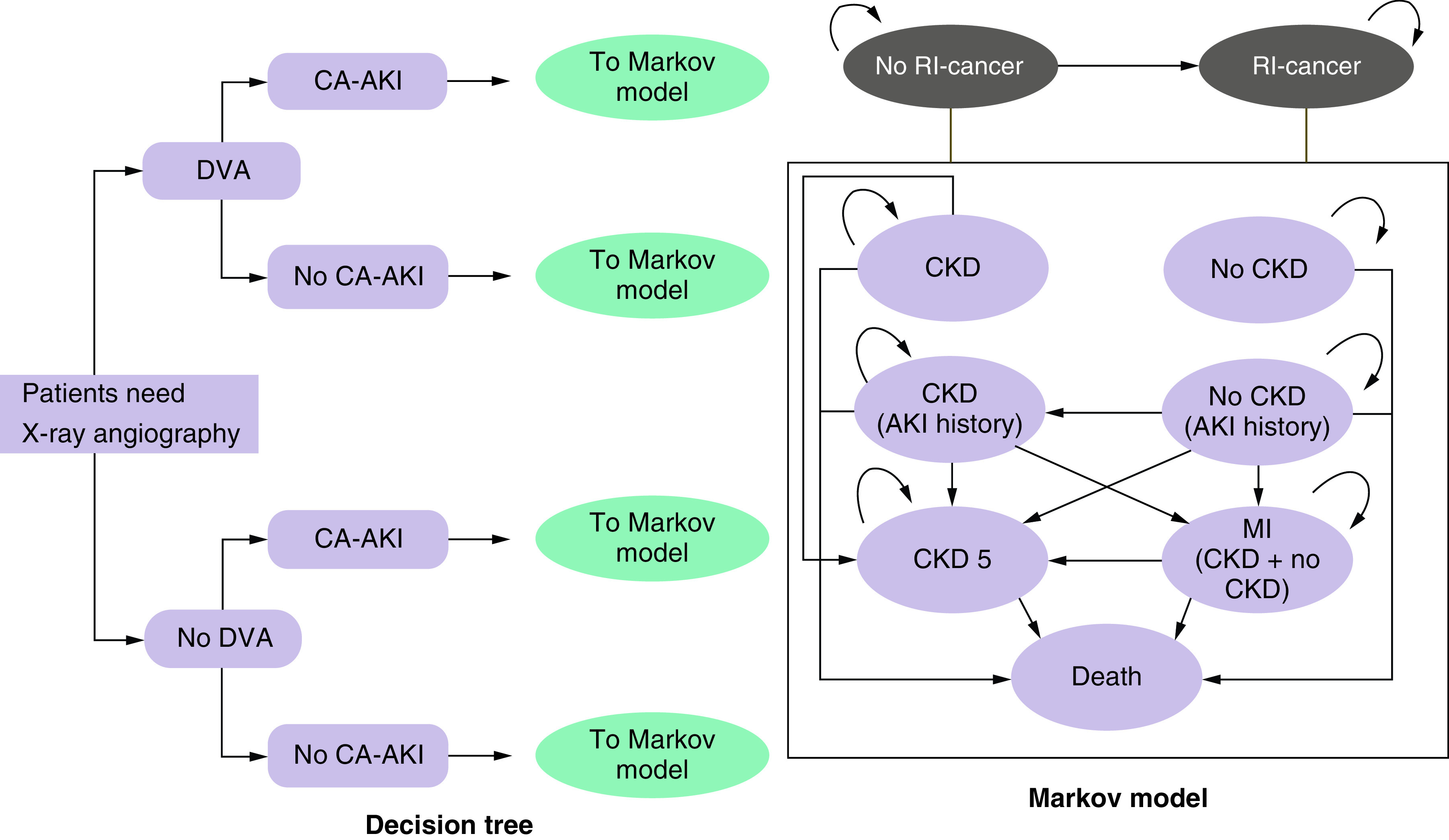
Decision tree and Markov model structure. CA-AKI: Contrast-associated acute kidney injury; CKD: Chronic kidney disease; DVA: Digital variance angiography; RI: Radiation-induced.

The economic model structure is presented in Figure 1. A two-part economic model was developed consisting of a decision tree to capture the short-term costs and outcomes associated with the respective image processing methods, and a Markov model to capture longer-term effects, with the potential for patients to transition between individual health states over time.

In the decision tree model, patients initially undergo x-ray angiography, with image processing based on current practice. Following the initial procedure, patients may either incur a CA-AKI or not. In the event that a CA-AKI is incurred, patients undergo further treatment. Patients may then either experience CKD or not, at which point they enter the Markov model. Preventive measures, such as minimizing contrast volume, discontinuing nephrotoxic medications and ensuring adequate intravascular volume expansion, play a crucial role in mitigating the risk of CA-AKI and its potential long-term consequences [15].

The starting health state in the Markov model is determined by whether or not patients have experienced CKD (i.e., patients begin in a ‘CKD’ health state or a ‘No CKD’ health state). Additionally, patients are categorized in the Markov model according to whether they have a history of CA-AKI. The previous occurrence of AKI is considered in the Markov model in terms of its impact on costs and clinical outcomes, with health states capturing AKI history utilized to capture this impact. Patient's progression through the Markov model is determined by the natural disease progression of CKD, as well as whether or not they have experienced AKI and the potential occurrence of cardiovascular events, in other words, MI. Patients beginning with CKD may remain in their initial health state, progress to more advanced stages (stratified according to previous occurrence of AKI) or experience death in any model cycle. Patients without CKD may also remain in their initial health state or progress to CKD in any model cycle, as well as experience death. Patients with non-severe renal insufficiency (i.e., early-stage CKD) may experience an MI at any point over the model time horizon. Patients who enter the CKD stage 5 health state, which is the most severe stage of CKD, are assumed to either remain in this health state or die, as they will be receiving dialysis and treatments to prevent an MI. The potential for health state regression, in other words, transition of patients to less severe health states, is not considered in the model.

‘Death’ is an absorbing health state, which tracks the number of patient deaths over the model time horizon. Patients are at risk of death from all causes during any given model cycle, with the risk of death conditional on stage of CKD, history of AKI and/or MI, occurrence of cancer, as well as age and sex of the individual patient. Age- and sex-specific mortality rates were derived from general population mortality statistics reported in national life tables in England [16].

The model also captures overall costs and quality-adjusted life-years (QALYs) associated with RI cancer, and the impact that a reduction in radiation exposure through use of the intervention may have on these costs and outcomes. It is assumed that no patients in the model have RI cancer at the beginning of the analysis, with the potential for cancer to develop in the years following initial exposure to radiation during imaging. The development of cancer is assumed to have no impact on CKD progression, including the occurrence of CKD and related cardiovascular events (MI), and therefore its development does not affect the transition probabilities associated with CKD. This component of the analysis allows for a thorough assessment of the potential benefits associated with image processing using DVA, including consideration of the impact that a reduction in radiation exposure, and CM volume, may have on overall costs and clinical outcomes.

At model entry, patients are assumed to be an average starting age of 68 (71% male), based on pooled patient data from Yuan *et al.*, which looked at safe contrast limits for the prevention of AKI following percutaneous coronary intervention (PCI) [17]. Further detail related to the modeling methodology, including a description of all model parameters, is presented in Section 2.2.

### Model inputs

The clinical, utility and cost inputs that were utilized in the economic model are presented in the following section. For a comprehensive overview of all model input parameters, including a description of parameter distributions, please refer to Table 1.

**Table 1.** Model input parameters.

Study	Parameter	Base-case value	Distribution	Distribution parameter	Sensitivity analysis range (low–high value)	Ref.
Clinical inputs						
**Baseline population characteristics**						
Yuan *et al.*, 2021 (pooled data)	Start age, years	68	Normal (μ, σ)	μ = -297,539.60, σ = 293,164.02	66–70	[17]
Yuan *et al.*, 2021 (pooled data)						
	Proportion of male patients	0.71	Beta (α, β)	α = 18,585.81, β = 7418.72	0.71–0.72	[17]
Meta-analysis; Dangas *et al.* (2005), Mehran *et al.* (2004), Mueller *et al.* (2002)	Prevalence of CKD among patients who undergo PCI	0.26	Beta (α, β)	α = 45.61, β = 131.17	0.19–0.32	[18–20]
Yuan *et al.*, 2021 (pooled data)	BMI	28.79	Normal (μ, σ)	μ = -16,744,177.286, σ = 16,162,539.27	28.72–28.86	[17]
Yuan *et al.*, 2021 (pooled data)	IABP before procedure	0.003	Beta (α, β)	α = 87.18, β = 25,969.07	0.003–0.004	[17]
Yuan *et al.*, 2021 (pooled data)	CrCl	77.75	Log Normal (μ, σ)	μ = 4.35, σ = 0.002	77.42–78.08	[17]
Yuan *et al.*, 2021 (pooled data)	CrCl^2^	6045.03	Log Normal (μ, σ)	μ = 8.71, σ = 0.004	5993.29–6096.99	[17]
Yuan *et al.*, 2021 (pooled data)	CrCl^3^	469,999	Log Normal (μ, σ)	μ = 13.06, σ = 0.007	463,978.27–476,072.57	[17]
Yuan *et al.*, 2021 (pooled data)	Log (Pre-procedure Hgb)	2.59	Beta (α, β)	α = -67,074.15, β = 41,154.42	2.56–2.61	[17]
Yuan *et al.*, 2021 (pooled data)	Diabetes	0.38	Beta (α, β)	α = 9886.92, β = 16,117.50	0.37–0.39	[17]
Yuan *et al.*, 2021 (pooled data)	Hypertension	0.81	Beta (α, β)	α = 20,996.26, β = 5008.54	0.80–0.81	[17]
Yuan *et al.*, 2021 (pooled data)	History of heart failure	0.17	Beta (α, β)	α = 24,370.49, β = 21,634.44	0.16–0.17	[17]
Yuan *et al.*, 2021 (pooled data)	Pre-procedure cardiogenic shock	0.03	Beta (α, β)	α = 830.24, β = 25,179.12	0.03–0.03	[17]
Yuan *et al.*, 2021 (pooled data)	NYHA class last 2 weeks (1–4)	1.35	Beta (α, β)	α = -8847.96, β = 2286.87	1.33–1.37	[17]
Yuan *et al.*, 2021 (pooled data)	History of MI	0.27	Beta (α, β)	α = 6891.68, β = 19,112.89	0.26–0.27	[17]
Yuan *et al.*, 2021 (pooled data)	History of PCI	0.35	Beta (α, β)	α = 9208.87, β = 16,795.58	0.35–0.36	[17]
Yuan *et al.*, 2021 (pooled data)	History of CABG	0.14	Beta (α, β)	α = 3699.44, β = 22,305.67	0.14–0.15	[17]
Yuan *et al.*, 2021 (pooled data)	History of CVD	0.13	Beta (α, β)	α = 3396.42, β = 22,608.80	0.13–0.13	[17]
Yuan *et al.*, 2021 (pooled data)	History of PAD	0.10	Beta (α, β)	α = 2711.37, β = 23,294.18	0.10–0.11	[17]
Yuan *et al.*, 2021 (pooled data)	History of chronic lung disease	0.12	Beta (α, β)	α = 3077.40, β = 22,927.96	0.11–0.12	[17]
Yuan *et al.*, 2021 (pooled data)	NSTEMI or UA	0.61	Beta (α, β)	α = 15,874.47, β = 10,129.94	0.60–0.62	[17]
Yuan *et al.*, 2021 (pooled data)	STEMI	0.20	Beta (α, β)	α = 5217.56, β = 20,787.21	0.20–0.21	[17]
Yuan *et al.*, 2021 (pooled data)	Cardiac arrest in last 24 h	0.03	Beta (α, β)	α = 786.23, β = 25,223.43	0.03–0.03	[17]
Yuan *et al.*, 2021 (pooled data)	CA-AKI	0.10	Beta (α, β)	α = 2590.36, β = 23,415.27	0.10–0.10	[17]
**CM volume and radiation exposure**						
Gyánó *et al.* (2019)	CM volume (ml) – No DVA	78	Log Normal (μ, σ)	μ = 4.36, σ = 0.04	72.25–83.75	[10]
Orias *et al.* (2020)	Reduction in CM – DVA (%)	0.50	Beta (α, β)	α = 48.02, β = 48.02	0.40–0.60	[21]
Juszkat *et al.* (2009), Gyánó *et al.* (2021)	Radiation exposure in no DVA (unit Gy)	0.80	Log Normal (μ, σ)	μ = -0.23, σ = 0.03	0.75–0.85	[1,22]
Gyánó *et al.* (2021)	Relative reduction in radiation exposure with DVA	0.70	Beta (α, β)	α = 28.81, β = 12.35	0.56–0.84	[1]
**Patient transition probabilities and HRs**						
Calculation – Yuan *et al.* (2021)	Probability of CA-AKI after PCI	0.04				[17]
Feldman *et al.* (2019)	Proportion of CKD 3–4 among CKD patients	27.71%	Beta (α, β)	α = 1,011,586.62, β = 2,638,882.13	27.66%–27.75%	[23]
Feldman *et al.* (2019)	Proportion of CKD 5 among CKD patients	3.277%	Beta (α, β)	α = 119,610.60, β = 3,530,862.81	3.258%–3.295%	[23]
James *et al.* (2010)	CA-AKI to CKD 5	3.280%	Beta (α, β)	α = 1,223.76, β = 36,380.91	3.100%–3.460%	[24]
Eriksen *et al.* (2006) & NICE CG169	CKD 3–4 to CKD 5 <69 years	0.018%	Beta (α, β)	α = 96.02, β = 533,363.49	0.0144%–0.0216%	[25,26]
Eriksen *et al.* (2006) & NICE CG169	CKD 3–4 to CKD 5 70–79 years	0.1000%	Beta (α, β)	α = 95.94, β = 95,848.02	0.0800%–0.1200%	[25,26]
Eriksen *et al.* (2006) & NICE CG169	CKD 3–4 to CKD 5 >79 years	0.0800%	Beta (α, β)	α = 95.96, β = 119,858.00	0.0640%–0.0960%	[25,26]
Coca *et al.* (2012)	HR of CKD for patients with AKI history	8.82	Log Normal (μ, σ)	μ = 2.00, σ = 0.59	3.05–25.48	[27]
**MI and AKI event probabilities and HRs**						
Valle *et al.* (2017)	Risk of MI, acute phase (with previous CA-AKI)	2.58%	Beta (α, β)	α = 458.29, β = 17,294.54	2.35%–2.82%	[28]
Valle *et al.* (2017)						
	Risk of MI, subsequent (with previous CA-AKI)	1.23%	Beta (α, β)	α = 2265.70, β = 181,392.89	1.18%–1.28%	[28]
Calculation - Yuan *et al.* (2021)	RR reduction in CA-AKI associated with DVA	0.08				[17]
Valle *et al.* (2017)	Risk of AKI requiring dialysis, acute phase (with previous CA-AKI)	0.79%	Beta (α, β)	α = 120.56, β = 15,183.08	0.65%–0.93%	[28]
Valle *et al.* (2017)	Risk of AKI requiring dialysis, subsequent (with previous CA-AKI)	0.16%	Beta (α, β)	α = 392.95, β = 238,351.60	0.15%–0.18%	[28]
Valle *et al.* (2017)	Risk of AKI requiring dialysis, subsequent (without previous CA-AKI)	0.04%	Beta (α, β)	α = 464.87, β = 1,267,580.11	0.0367%–0.0433%	[28]
Valle *et al.* (2017)	Probability of MI with AKI history	0.671%	Beta (α, β)	α = 95.40, β = 14,120.00	0.537%–0.805%	[28]
Valle *et al.* (2017)	Probability of MI without AKI history	0.495%	Beta (α, β)	α = 95.56, β = 19,202.64	0.396%–0.594%	[28]
Smolina *et al.* (2012)	Probability of MI post MI	1.234%	Beta (α, β)	α = 94.86, β = 7594.15	0.987%–1.480%	[29]
**Annual probabilities and HRs or RR of death**						
Valle *et al.* (2017)	HR of CA-AKI to death	2.13	Log Normal (μ, σ)	μ = 0.76, σ = 0.03	2.01–2.26	[28]
NICE MTG60	CKD 3–4 to death RR (conditional on age and sex) male <69 years	3.60	Log Normal (μ, σ)	μ = 1.27, σ = 0.17	2.60–5.00	[30]
NICE MTG60	CKD 3–4 to death RR (conditional on age and sex) female <69 years	2.70	Log Normal (μ, σ)	μ = 0.98, σ = 0.16	2.00–3.70	[30]
NICE MTG60	CKD 3–4 to death RR (conditional on age and sex) male 70–79 years	2.40	Log Normal (μ, σ)	μ = 0.87, σ = 0.10	2.00–2.90	[30]
NICE MTG60	CKD 3–4 to death RR (conditional on age and sex) female 70–79 years	1.80	Log Normal (μ, σ)	μ = 0.58, σ = 0.08	1.50–2.10	[30]
NICE MTG60	CKD 3–4 to death RR (conditional on age and sex) male >79 years	2.30	Log Normal (μ, σ)	μ = 0.83, σ = 0.07	2.00–2.60	[30]
NICE MTG60	CKD 3–4 to death RR (conditional on age and sex) female >79 years	2.10	Log Normal (μ, σ)	μ = 0.74, σ = 0.05	1.90–2.30	[30]
Villar *et al.* (2007)	CKD 5 to death RR (conditional on age and sex) male 18–64 years	10.00	Log Normal (μ, σ)	μ = 2.29, σ = 0.17	7.10–13.70	[31]
Villar *et al.* (2007)	CKD 5 to death RR (conditional on age and sex) female 18–64 years	16.40	Log Normal (μ, σ)	μ = 2.77, σ = 0.26	9.60–26.30	[31]
Villar *et al.* (2007)	CKD 5 to death RR (conditional on age and sex) male >64 years	4.80	Log Normal (μ, σ)	μ = 1.56, σ = 0.10	3.90–5.80	[31]
Villar *et al.* (2007)	CKD 5 to death RR (conditional on age and sex) female >64 years	7.10	Log Normal (μ, σ)	μ = 1.95, σ = 0.14	5.40–9.20	[31]
NICE TA236	MI (acute) to death SMR	5.84	Log Normal (μ, σ)	μ = 1.76, σ = 0.13	4.38–7.30	[32]
NICE TA236	MI (subsequent) to death SMR	2.21	Log Normal (μ, σ)	μ = 0.76, σ = 0.13	1.66–2.76	[32]

Utility inputs						
**Utilities – baseline**						
Jesky *et al.* (2016)	No CKD	0.85	Beta (α, β)	α = 26.65, β = 4.70	0.75–1.00	[33]
Jesky *et al.* (2016)	CKD stage 3	0.80	Beta (α, β)			
				α = 175.58, β = 43.90	0.68–1.00	[33]
Jesky *et al.* (2016)	CKD stage 4	0.74	Beta (α, β)	α = 322.85, β = 113.43	0.62–0.85	[33]
Jesky *et al.* (2016)	CKD stage 5 – pre-dialysis	0.73	Beta (α, β)	α = 132.12, β = 48.87	0.62–1.00	[33]
Lee *et al.* (2005)	CKD stage 5 –dialysis	0.53	Beta (α, β)	α = 181.10, β = 160.60	0.26–0.80	[34]
Cooper *et al.* (2020)	Myocardial infarction acute effect (weeks 1–13)	0.52	Beta (α, β)	α = 294.31, β = 271.67	0.47–0.58	[35]
Cooper *et al.* (2020)	Myocardial infarction effect (>week 13)	0.66	Beta (α, β)	α = 136.0, β = 70.06	0.57–0.76	[35]
Sullivan *et al.* (2011)	CA-AKI	0.52	Beta (α, β)	α = 120.02, β = 109.02	0.46–0.59	[36]
**Disutility values** ^**†**^						
EOS 2D/3D x-ray Imaging System – Final Report 2011	Disutility: breast cancer	3.42 (QALYS lost distributed proportionally)	Normal (μ, σ)	μ = -38,901.23, σ = 27,532.92	2.74–4.11	[37]
EOS 2D/3D x-ray Imaging System – Final Report (2011)	Disutility: lung cancer	6.80 (QALYS lost distributed proportionally)	Normal (μ, σ)	μ = -608,457.27, σ = 518,992.73	5.44–8.16	[37]
EOS 2D/3D x-ray Imaging System – Final Report (2011)	Disutility: colorectal cancer	3.45 (QALYS lost distributed proportionally)	Normal (μ, σ)	μ = -26,759.38, σ = 19,001.46	2.76–4.14	[37]
EOS 2D/3D x-ray Imaging System – Final Report (2011)	Disutility: prostate cancer	4.62 (QALYS lost distributed proportionally)	Normal (μ, σ)	μ = -106,185.57, σ = 83,214.60	3.70–5.55	[37]

Cost inputs (excluding DVA-related)						
NHS Reference Costs 2020	CKD stage 3–4	£271	Gamma	α = 384.16, β = 0.71	244–299	[38]
NHS Reference Costs 2020						
	CKD stage 5 first cycle	£7111	Gamma	α = 384.16, β = 18.51	6400–7822	[38]
NHS Reference Costs 2020	CKD stage 5 subsequent cycles	£6170	Gamma	α = 384.16, β = 16.06	5553–6787	[38]
Walker *et al.* 2016	Cost of MI (initial)	£6275	Gamma	α = 7703.04, β = 0.81	6135–6415	[39]
Walker *et al.* 2016	Cost of MI (subsequent)	£573	Gamma	α = 423.67, β = 1.35	518–628	[39]
NHS Reference Costs 2020	CA-AKI: cost of index admission	£1961	Gamma	α = 384.16, β = 5.11	1765–2157	[38]
NHS Reference Costs 2020	CA-AKI: cost of hospital day	£367	Gamma	α = 384.16, β = 0.95	330–403	[38]
Calculation	CA-AKI: cost of extended hospital admission	£1375				
EOS 2D/3D x-ray Imaging System – Final Report 2011	Cost of breast cancer^‡^	£17,224	Gamma	α = 15.37, β = 1,120.92	12,918–30,143	[37]
EOS 2D/3D x-ray Imaging System – Final Report 2011	Cost of lung cancer^‡^	£28,089	Gamma	α = 15.37, β = 1,827.98	21,067–49,157	[37]
EOS 2D/3D x-ray Imaging System – Final Report 2011	Cost of colorectal cancer^‡^	£17,408	Gamma	α = 15.37, β = 1,132.83	13,056–30,463	[37]
EOS 2D/3D x-ray Imaging System – Final Report 2011	Cost of prostate cancer^‡^	£15,322	Gamma	α = 15.37, β = 997.13	11,492–26,814	[37]

Resource use-related information						
Assumption	Extended hospital admissions compared with new admissions (%)	0.50	Normal	μ = 48.02, σ = 48.02	0.40–0.60	
Subramanian *et al.* (2007), Kerr *et al.* (2014)	Extra hospital bed days due to CA-AKI	3.75	Log Normal	μ = 1.32, σ = 0.10	3.00–4.50	[40,41]

Cost inputs (DVA-related)						
Tripathi *et al.* (2021)	Procedure time (min)	30	Log Normal	μ = 3.40, σ = 0.00	0.00–0.00	[42]
Assumption	Preparation time (min)	10	Log Normal	μ = 2.30, σ = 0.10	8.00–12.00	
King's Health Partners collaboration https://rb.gy/0sqctz	Cath lab availability h per week	72	Log Normal	μ = 4.27, σ = 0.10	57.60–86.40	[43]
King's Health Partners collaboration https://rb.gy/0sqctz	Cath lab available weeks per year	48	Log Normal	μ = 3.87, σ = 0.10	38.40–57.60	[43]
https://www.work-day.co.uk/	Cath lab available h per year	2765	Fixed			[44]
King's Health Partners collaboration https://rb.gy/0sqctz	Utilization of cath lab for non-priority cases	0.8	Beta	α = 19.21, β = 4.80	0.64–0.96	[43]
Calculation (base-case)	Maximum number of angiography cases per year	4147				
Fletcher *et al.* (2017)	Cath lab unit cost	22	Gamma	α = 96.04, β = 0.23	17.70–26.55	[45]
Assumption	Cath lab overhead costs (proportion)	0.20	Beta	α = 76.83, β = 307.33	0.16–0.24	
Kinepict Health	Cost of DVA hardware, monitor, and installation	£29,200	Fixed			
Kinepict Health	Device lifespan (years)	5	Fixed			
Kinepict Health	Annual maintenance costs	£35,800	Fixed			

† Disutilities assigned across the entire proportion of patients developing the different forms of cancer.

‡ Cancer costs assigned proportionally.

AKI: Acute kidney injury; BMI: Body mass index; CABG: Coronary artery bypass graft; CA-AKI: Contrast-associated acute kidney injury; CKD: Chronic kidney disease; CM: Contrast media; CrCl: Creatinine clearance; CVD: Cardiovascular disease; DVA: Digital variance angiography; Gy: Gray; Hgb: Hemoglobin; HR: Hazard ratio; IABP: Intra-aortic balloon pump; MI: Myocardial infarction; NSTEMI: Non-ST-elevation myocardial infarction; NYHA: New York Heart Association; PAD: Peripheral arterial disease; PCI: Percutaneous coronary intervention; RR: Relative risk; SMR: Standardized mortality ratio; UA: Unstable angina.

#### Clinical effectiveness parameters

In the absence of specific data for patients undergoing lower limb x-ray angiography, a study by Yuan *et al*, which was based on patients who underwent PCI, was used to estimate the probability of CA-AKI following intervention [17]. Therefore, it was assumed that the baseline patient characteristics in our model, including data on clinical history and presentation, would be identical to the patient characteristics reported in Yuan *et al.* (pooled data). For the initial imaging procedure, the volume of CM used per procedure when undergoing imaging using the comparator (i.e., current practice) was estimated to be 78 ml, based on data from Gyánó *et al.*, which looked at the average CM used for 42 patients undergoing lower limb x-ray angiography [10]. A probability of 4.42% was estimated for the occurrence of CA-AKI among patients undergoing current practice imaging in the base-case analysis, based on data from the ‘full model’ in Yuan *et al.* [17]. This study explored the risk of AKI based on logistic regression models developed to predict the risk of CA-AKI, using a prospective sample of 5423 PCI procedures for model validation. The base-case value used in our analysis (4.42%) was based on what Yuan *et al.* described as a ‘full model’, which utilized patient data on a range of different risk factors, to predict CA-AKI risk. Alternative models were also explored in this study to estimate alternative risk values based on the selected risk factors included in the analysis. In Yuan *et al.*, these alternative models were described as a ‘pragmatic full model’, and a ‘pragmatic minimum model’, and both produced alternative estimates of risk based on included risk factors [17]. A complete overview of the risk factors included in the three models performed by Yuan *et al.* is presented in Table 2. These alternative values for the occurrence of CA-AKI (4.99% in the pragmatic full model, and 5.24% in the pragmatic minimum model) were explored in sensitivity analysis [17]. A short summary of the formulae used in the calculation of these values is presented in Supplementary Materials.

**Table 2.** Predictors used for each CA-AKI prediction model in Yuan *et al.* 2021.

Full model	Pragmatic full model	Pragmatic minimum model
Contrast volume	Contrast volume	Contrast volume
Age	Age	Age
Sex	Sex	Sex
BMI	BMI	BMI
Intra-aortic balloon pump before procedure	Intra-aortic balloon pump before procedure	Intra-aortic balloon pump before procedure
Creatinine clearance	Creatinine clearance	Creatinine clearance
Pre-procedure hemoglobin	Pre-procedure hemoglobin	Pre-procedure hemoglobin
History of diabetes mellitus	History of diabetes mellitus	
History of hypertension	History of hypertension	
History of heart failure	History of heart failure	
Pre-procedure cardiogenic shock	Pre-procedure cardiogenic shock	
Heart failure symptoms in past 2 weeks		
History of myocardial infarction		
History of percutaneous coronary intervention		
History of coronary artery bypass graft		
History of cardiovascular disease		
History of peripheral artery disease		
History of chronic lung disease		
Coronary artery disease presentation (Unstable angina, non-ST-elevation myocardial infarction, ST-elevation myocardial infarction)		
Cardiac arrest in past 24 h		

Data taken from [17].

Data from Orias *et al.* were used to estimate the relative reduction in the volume of CM used with image processing using DVA (50%) [21], with a relative risk (RR) reduction (8.23%) in the occurrence of CA-AKI with DVA subsequently estimated in the base-case analysis, based on data from the full model in Yuan *et al.* [17]. As with the base-case occurrence of CA-AKI, alternative values for the risk reduction attributable to DVA from the pragmatic full (7.27%) and pragmatic minimum (7.06%) models from Yuan *et al.* were also explored in sensitivity analysis [17].

The amount of radiation exposure when undergoing imaging with current practice was estimated using data from previous literature (0.80 gray [Gy]) [1,22]. Information from Gyánó *et al.* was used to estimate the relative 70% reduction in the risk of radiation exposure with DVA [1], which was then used to inform the subsequent reduction in the risk of cancer, with DVA. The methodology applied to estimate these values was informed by prior work conducted as part of the York radiation modeling study. The York modeling study reported on the risk of all cancers (per unit Gy) among males and females up to the age of 99 (varying risk depending on age category) [46,47]. These values were used to estimate the cancer risk (for breast cancer, lung cancer, colorectal cancer and prostate cancer) attributable to radiation exposure for patients in the current practice arm of the model. Using this information in combination with data on the relative reduction in the risk of radiation exposure with DVA from Gyánó *et al.* [1], the reduction in the risk of each type of cancer, outlined above, with use of DVA was estimated. Based on this methodology, the reductions in the risk of the various types of cancer, as well as the impact that this would have on cancer-related costs and QALYs, were estimated.

The proportion of CKD patients with CKD stages 3–4 and stage 5 was informed by information from Feldman *et al.* [23], which assessed the rates of drug eluting stent (DES) usage in patients with CKD between 2009 and 2015. The transition probabilities for patients experiencing CA-AKI to the latter stage health states, as well as for patients with stage 3–4 CKD progressing to stage 5 at different age categories, were sourced from prior literature [24–26]. The hazard ratio (HR) of experiencing CKD among patients without CKD but with a prior history of AKI was sourced from a study by Coca *et al.* [27]. The probability of MI with (0.67%), and without (0.50%), AKI history were sourced from a study by Valle *et al.*, which looked at the risk of adverse events in patients with AKI following PCI procedures [28]. Further information on the risk of MI and AKI, with and without history of CA-AKI, as well as the risk reduction of CA-AKI associated with DVA utilized in the economic model, are presented in Table 1. Additionally, HRs and RR of death associated with experiencing CA-AKI or MI are presented, as well as the RR of death associated with occupying the various CKD health states. A complete overview of baseline patient characteristics, as well as all clinical data and transition probabilities utilized in the model, are presented in Table 1.

#### Utilities

Utility values were only utilized in the Markov component of the economic model. The utility values among patients without CKD, and with CKD stages 1–5 (up to pre-dialysis in CKD stage 5) were all derived from a previous prospective observational study exploring health-related quality-of-life (HRQoL) among a UK cohort of patients with CKD [33]. For post-dialysis patients in the CKD stage 5 health state, a utility value of 0.53 was sourced from a study by Lee *et al.*, which characterized and compared HRQoL among patients with renal failure [34]. For patients experiencing MI (both acute effect [weeks 1–13] and prolonged effect [>13 weeks]), utility values were sourced from a systematic review by Cooper *et al.*, which explored HRQoL weights for economic evaluation through different stages of CKD [35]. The utility value of patients experiencing CA-AKI (0.66) was sourced from this same review [35]. Disutility values for patients developing RI cancer were also included in the model. Separate disutility values for the following cancer types were included in the model: breast, lung, colorectal and prostate. These disutility values were estimated from a previous NICE diagnostic assessment report on the EOS 2D/3D x-ray imaging system [37]. Disutility values were applied in the model by estimating the QALYs lost with the various forms of cancer, based on data from the NICE report [37], and then proportionally distributing the estimated disutility among the affected population [46,48]. All utility and disutility values included in the model are presented in Table 1.

#### Costs

The following information was used in the calculation of the per procedure additional cost associated with use of DVA: hardware, monitor and installation costs, device lifespan, procedure time, preparation time, availability of catheterization laboratory per year (hourly) and associated costs, including overheads, as well as the annual maintenance and warranty costs associated with the DVA device. This information was used to calculate a per procedure average cost associated with DVA (£10.19; SD = 1.91, min = 6.41, max = 18.16), which considered the overall cost of DVA and related resources, as well as the maximum number of angiography procedures that would be performed per year (estimated at 4147, considering the available cath lab h per year [43,44], as well as the angiography preparation and procedure time [42]). Therefore, in the intervention arm, use of DVA alongside current practice imaging was assumed to be acceptable in 100% of cases. This cost was applied in the decision tree component of the economic model, at the point where patients would undergo initial imaging in the intervention arm of the analysis. A detailed overview of the parameters that were used in the calculation of DVA procedure costs, as well as the data sources and parameter distributions, is presented in Table 1.

The cost of CKD among patients in stages 3–4 was estimated utilizing relevant NHS resource use data sourced from the NHS Reference Costs, 2020 [38], as well as necessary medication costs sourced from the British National Formulary, 2020 [49]. A per cycle cost of £271 was estimated. The cost of CKD stage 5 for the first model cycle (£7111) was estimated using the same sources as described above, with the cost of CKD stage 5 from cycle 2 onwards (£6170) estimated based on the proportion of this population that would be undergoing conservative management, and the proportion that would be undergoing treatment for renal replacement therapy (RRT). The cost of the index admission for CA-AKI (£1961) was estimated using pooled data from the NHS Reference Costs, 2020 related to AKI, while the cost of an extra hospital day associated with AKI (£367) was also sourced from the NHS Reference Cost list [38]. A total cost of £1375 was estimated for extended hospital admission due to CA-AKI, based on the additional number of hospital bed days that would be required (3.75). This information on additional bed days required was sourced from a prior study by Subramanian *et al.*, which looked at the economic burden associated with contrast-induced nephropathy [40]. Costs associated with acute MI in the first (£6275) and subsequent cycles (£573) were derived from a previous study by Walker *et al.* [39]. The individual resource use components which were included in the calculation of health state costs were informed by the cost analysis performed as part of NICE Clinical Guidelines 169 (acute kidney injury: prevention, detection and management) [50]. Finally, the costs associated with incurring breast, lung, colorectal and prostate cancers were estimated using data sourced from the NICE Diagnostic Assessment Report on the EOS 2D/3D x-ray imaging system [37], using a similar methodology as was applied for the distribution of RI cancer-related disutilities. A full breakdown of all health state costs is presented in Table 1.

### Analysis

#### Base-case analysis

A cost-utility analysis was performed, with the final results presented in terms of cost per QALY gained associated with introduction of the DVA image processing method alongside current practice. The base-case results present an overview of the incremental costs and QALYs associated with introduction of DVA (individual patient basis). In addition, the percentage change in the occurrence of the various forms of cancer are presented.

Results of the base-case analysis are presented probabilistically. The probabilistic analysis allows for quantification of the level of confidence in the results of the analysis, considering the uncertainty that exists in the model inputs. In order to consider this uncertainty, a Monte Carlo simulation analysis was performed, with 5000 simulations of the economic model, and relevant probabilistic output were produced. Probabilistic results show the probability of the intervention being cost effective and cost saving, respectively, and are also presented in the form of a cost–effectiveness plane, which shows a scatterplot of results based on individual model simulations. A willingness-to-pay (WTP) threshold of £20,000/QALY was applied in the base-case analysis.

#### One-way sensitivity analysis

In order to consider the uncertainty in individual model parameters, one-way deterministic sensitivity analyses (DSA) were performed. The purpose was to explore the impact that individual parameter variation would have on the overall results. The sensitivity analyses results are presented in the form of tornado diagrams, which show how impactful each parameter is on the net monetary benefit (NMB), incremental costs, and incremental QALYs associated with the introduction of DVA image processing alongside current practice. Each parameter was varied by a pre-defined range (increased and decreased by 25%) in the DSA, or alternatively variation was based on the 95% confidence interval range of the individual parameter.

#### Scenario analyses

A number of different scenario analyses were performed to explore alternative assumptions to the base-case data. Due to the uncertainty that surrounds the risk of CA-AKI associated with use of CM, including the risk reduction associated with a reduction in volume, various different analyses were performed utilizing alternative data sources. In the first, it was assumed that data from NICE MTG 60 (DyeVert System for reducing the risk of acute kidney injury in coronary and peripheral angiography) [30] would be utilized to estimate the risk of CA-AKI following x-ray angiography in the current practice arm of the model (8.72%), rather than data from Yuan *et al.* [17]. In the second scenario analysis, data from Gurm *et al.*, which looked at impact of contrast dose reduction on AKI incidence [51], were utilized in the estimation of the RR reduction of CA-AKI following use of DVA (18.59%), as opposed to the data from Yuan *et al.* which were included in the base-case analysis [17]. In the third, the aforementioned data from NICE MTG 60 [30] and from Gurm *et al.* [51] were used in combination. Two further analyses were performed in which data from the alternative pragmatic full model, and pragmatic minimum model, included in Yuan *et al.* were used to estimate the probability of CA-AKI after x-ray angiography in the current practice arm and the RR reduction of CA-AKI with use of DVA, respectively (as described in Section 2.2.1) [17]. The results of each scenario analysis are presented in the same format as those of the base-case analysis.

## Results

Results of the base-case analysis are presented in Section 3.1, followed by those of the DSA (3.2), and scenario analyses (3.3).

### Base-case analysis

Base-case probabilistic analysis results presented in Table 3 indicate that the introduction of DVA alongside current practice imaging results in an average cost saving of £309 per patient, when considering initial procedure costs as well as longer-term complication and cancer-related costs associated with each strategy. The introduction of DVA also results in an incremental QALY gain of 0.025 per patient. Therefore, use of DVA image processing is a ‘dominant’ strategy, meaning that it is less costly and more effective than current practice. Table 3 also presents the percentage reduction in the risk of various forms of cancer following the introduction of DVA, due to the associated reduction in radiation exposure.

**Table 3.** Base-case probabilistic results.

Base-case analysis (English NHS perspective)		
Introduction of DVA		
	DVA	Current practice
Average cost per patient	£4141	£4450
QALYs per patient	9.089	9.064
Incremental costs per patient	-£309	
Incremental QALYs per patient	0.025	
Incremental cost–effectiveness ratio (costs/QALY)	Dominant	
Probability of being cost effective	100%	
Probability of being cost saving	100%	
Reduction in risk of cancer with DVA:		
Breast cancer	19.97%	
Lung cancer	70.00%	
Colorectal cancer	70.00%	
Prostate cancer	50.03%	

DVA: Digital variance angiography; NHS: National Health Service; QALY: Quality-adjusted life-year.

**Figure 2.**
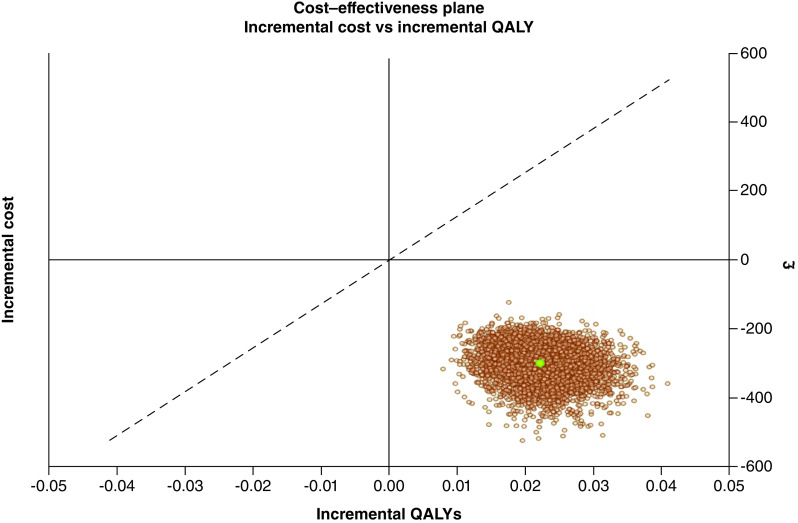
Incremental cost–effectiveness plane. Digital variance angiography versus no digital variance angiography: base-case probabilistic analysis. QALY: Quality-adjusted life-year.

Figure 2 (cost–effectiveness plane) presents further results of the probabilistic base-case analysis. Figure 2 indicates that in all model simulations, introduction of DVA is less costly and more effective than current practice. These results are also reflected in Table 3, where it is shown that introduction of DVA alongside current practice has a 100% probability of being cost effective and cost saving, respectively.

### One-way sensitivity analysis

**Figure 3.**
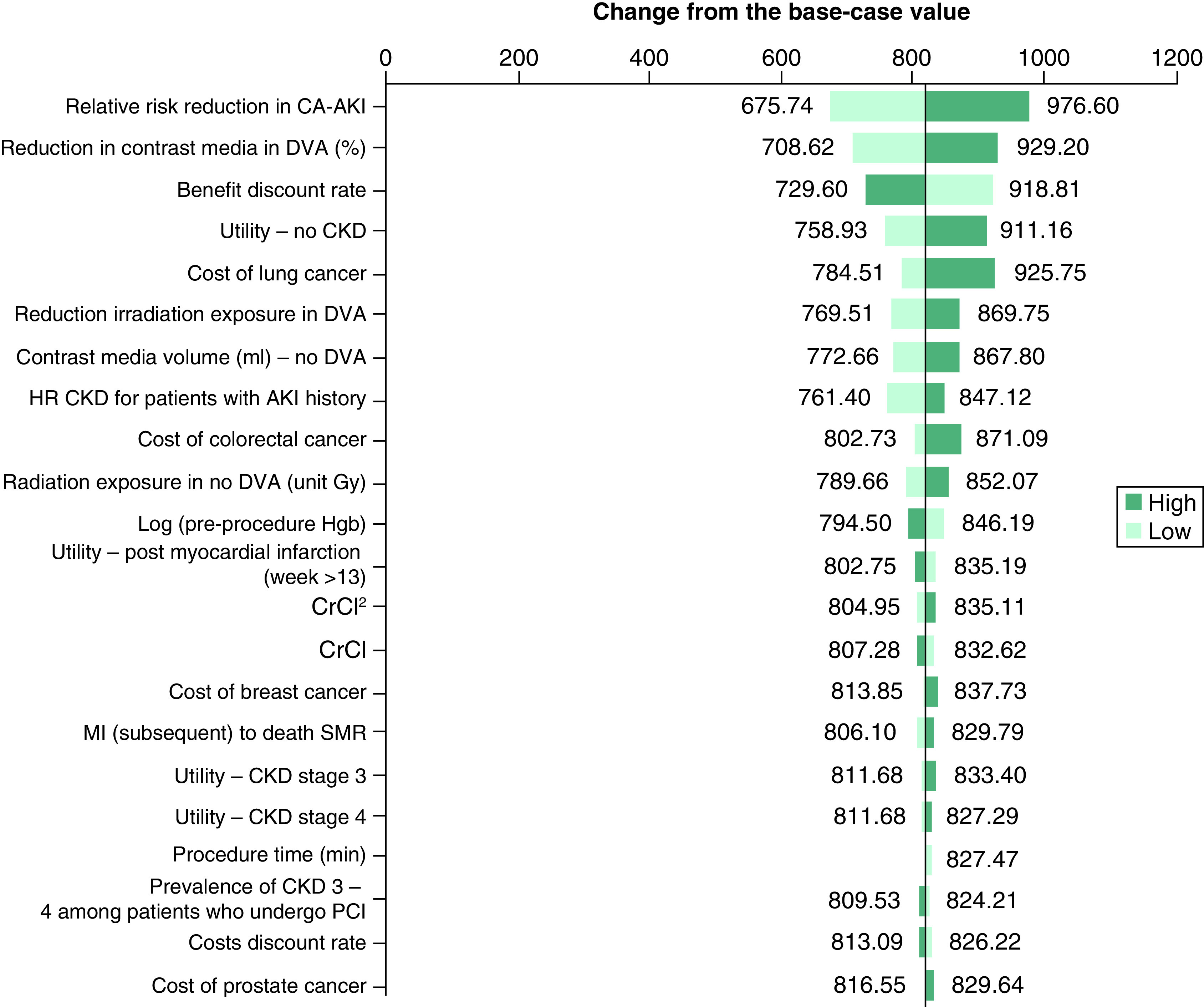
One-way sensitivity analysis (net monetary benefit) – tornado diagram. AKI: Acute kidney injury; CA-AKI: Contrast-associated acute kidney injury; CKD: Chronic kidney disease; DVA: Digital variance angiography; MI: Myocardial infarction; PCI: Percutaneous coronary intervention; SMR: Standardized mortality ratio.

**Figure 4.**
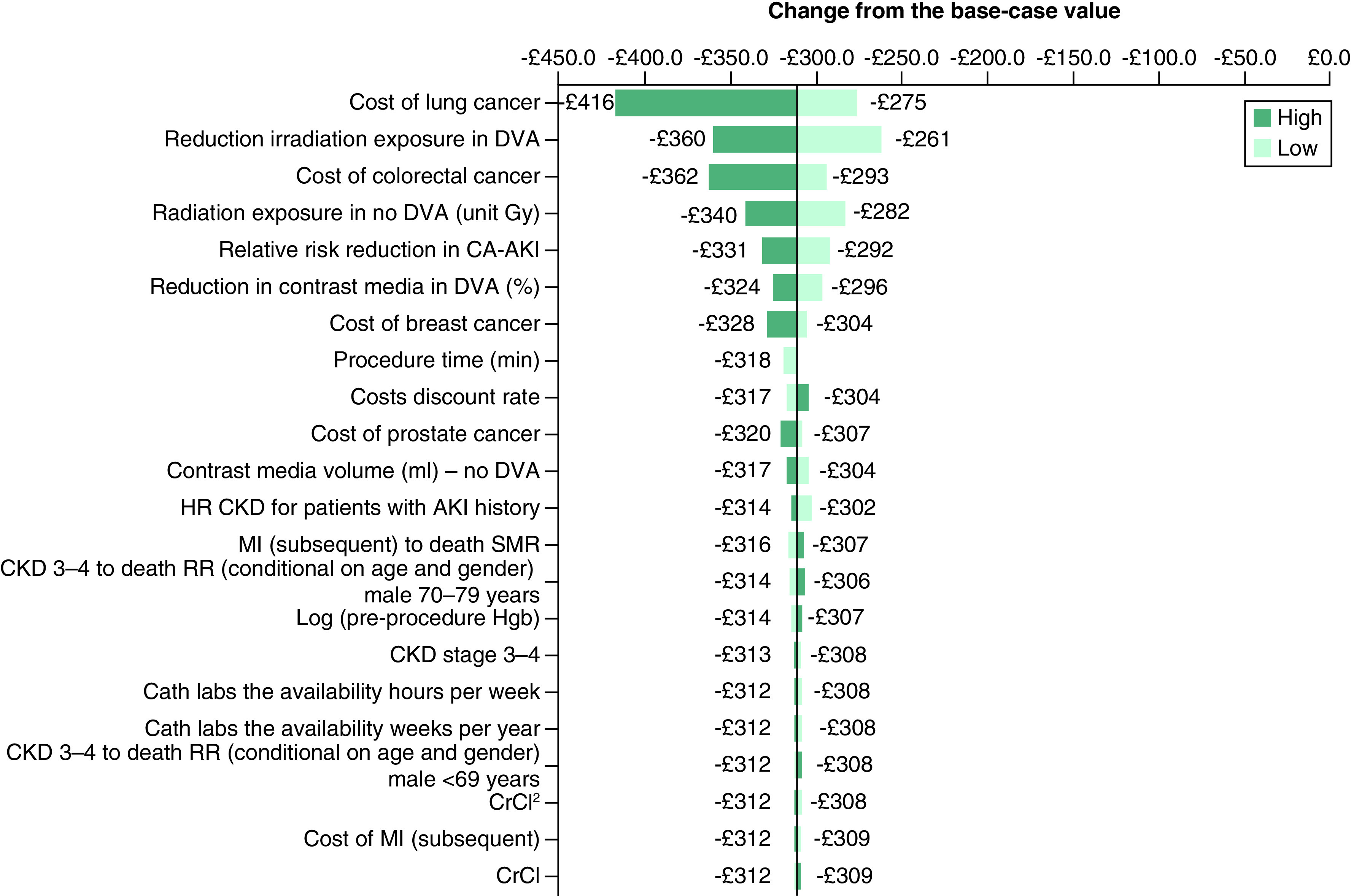
One-way sensitivity analysis (incremental cost) – tornado diagram. AKI: Acute kidney injury; CA-AKI: Contrast-associated acute kidney injury; CKD: Chronic kidney disease; DVA: Digital variance angiography; HR: Hazard ratio; MI: Myocardial infarction; RR: Relative risk; SMR: Standardized mortality ratio.

**Figure 5.**
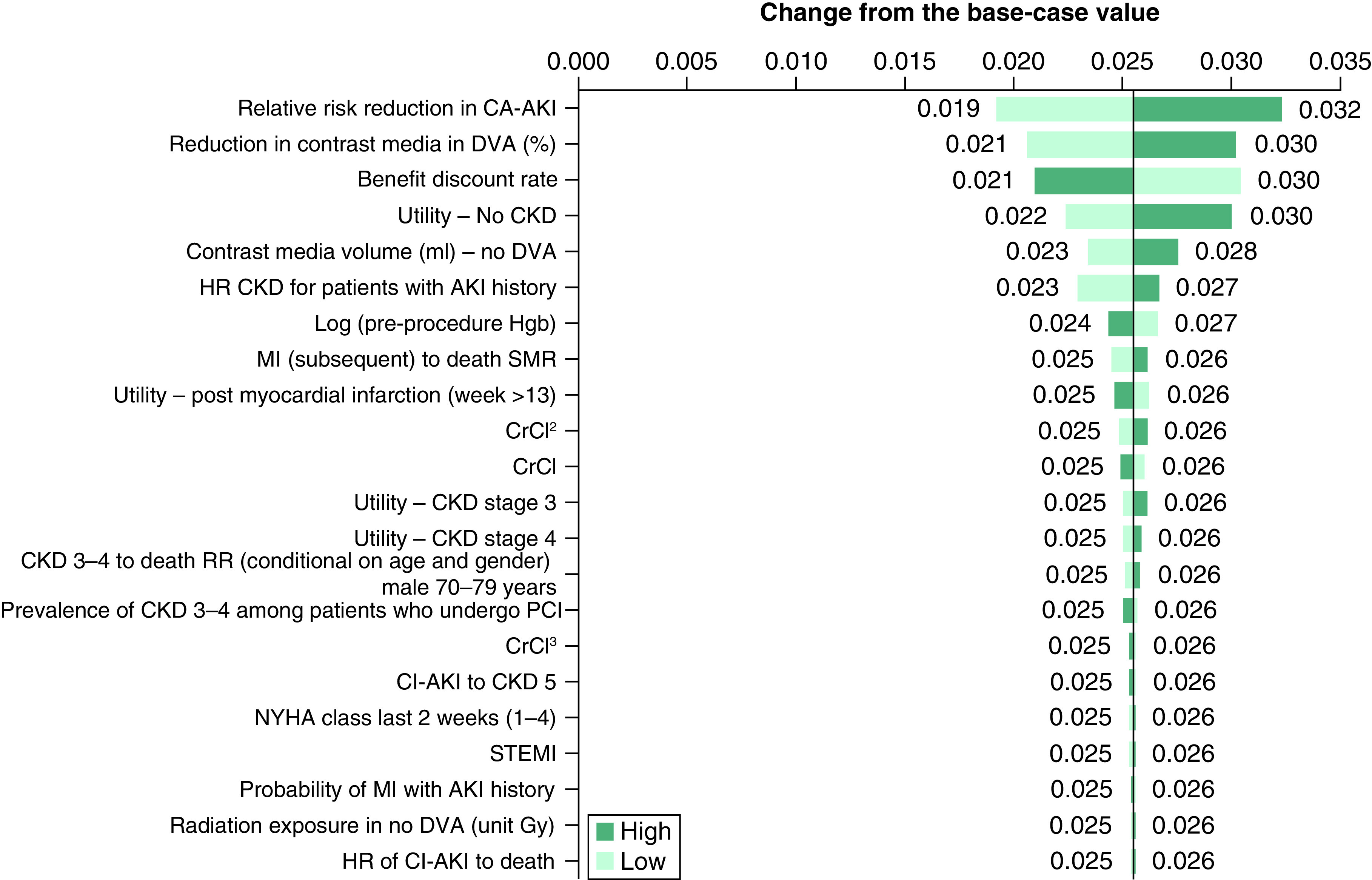
One-way sensitivity analysis (incremental quality-adjusted life-years) – tornado diagram. AKI: Acute kidney injury; CA-AKI: Contrast-associated acute kidney injury; CKD: Chronic kidney disease; DVA: Digital variance angiography; HR: Hazard ratio; NYHA: New York Heart Association; MI: Myocardial infarction; SMR: Standardized mortality ratio; STEMI: ST-elevation myocardial infarction.

Results of the one-way DSA are presented in Figure 3 (NMB; base-case analysis = £820), Figure 4 (incremental costs; base-case analysis = -£309), and Figure 5 (incremental QALYs; base-case analysis = 0.025) below. The most impactful parameters in the analysis of NMB (Figure 3) are the RR reduction in CA-AKI associated with the introduction of DVA, the percentage reduction in CM volume associated with DVA, and the utility value of No CKD. The same parameters are most impactful in the sensitivity analysis of incremental QALYs (Figure 5). Finally, in the analysis of incremental cost (Figure 4), the cost of lung cancer, the reduction in the risk of radiation exposure associated with introduction of DVA alongside current practice, and the cost of colorectal cancer, are most impactful on the results. Figure 4 shows that when the higher (increased) value for lung cancer is applied in the model, the incremental cost savings associated with DVA increase (-£416). Similar results are shown when the increased value for colorectal cancer is applied (-£362). Therefore, the potential for DVA image processing to reduce the occurrence of costly, cancerous events is a key driver of model results.

### Scenario analyses

Multiple individual scenario analyses were performed to explore alternative assumptions surrounding the base-case values applied in the model. As shown in Tables 4–6, when data from NICE MTG60 on the risk of CA-AKI following the initial imaging procedure [30], and from Gurm *et al.* on the risk reduction of CA-AKI with DVA image processing [51], were used in the analysis, cost savings and QALY gains associated with the intervention increased from the base-case analysis. Utilizing alternative model data from Yuan *et al.* [17] had little impact on the base-case results (Tables 7 & 8).

**Table 4.** Scenario analysis 1 probabilistic results.

Scenario analysis 1 – NICE MTG60 data used to estimate the risk of CA-AKI after PCI (8.72%) [30]		
Introduction of DVA		
	DVA	Current practice
Average cost per patient	£4914	£5295
QALYs per patient	8.783	8.735
Incremental costs per patient	-£381	
Incremental QALYs per patient	0.048	
Incremental cost–effectiveness ratio (costs/QALY)	Dominant	

CA-AKI: Contrast-associated acute kidney injury; DVA: Digital variance angiography; PCI: Percutaneous coronary intervention; QALY: Quality-adjusted life-year.

**Table 5.** Scenario analysis 2 probabilistic results.

Scenario analysis 2 – Gurm *et al.* (2016) data used to estimate the RR reduction in CA-AKI with DVA (18.59%) [51]		
Introduction of DVA		
	DVA	Current practice
Average cost per patient	£4073	£4472
QALYs per patient	9.149	9.093
Incremental costs per patient	-£399	
Incremental QALYs per patient	0.056	
Incremental cost–effectiveness ratio (costs/QALY)	Dominant	

CA-AKI: Contrast-associated acute kidney injury; DVA: Digital variance angiography; QALY: Quality-adjusted life-year; RR: Relative risk.

**Table 6.** Scenario analysis 3 probabilistic results.

Scenario analysis 3 – Scenario analyses 1 and 2 performed in combination		
Introduction of DVA		
	DVA	Current practice
Average cost per patient	£4732	£5285
QALYs per patient	8.878	8.769
Incremental costs per patient	-£553	
Incremental QALYs per patient	0.109	
Incremental cost–effectiveness ratio (costs/QALY)	Dominant	

DVA: Digital variance angiography; QALY: Quality-adjusted life-year.

**Table 7.** Scenario analysis 4 probabilistic results.

Scenario analysis 4 – Data from the Yuan *et al.* (2021) pragmatic full model used to estimate the probability of CA-AKI after PCI (4.99%) and the RR reduction in CA-AKI with DVA (7.27%) [17]		
Introduction of DVA		
	DVA	Current practice
Average cost per patient	£4269	£4579
QALYs per patient	9.066	9.041
Incremental costs per patient	-£310	
Incremental QALYs per patient	0.025	
Incremental cost–effectiveness ratio (costs/QALY)	Dominant	

CA-AKI: Contrast-associated acute kidney injury; DVA: Digital variance angiography; PCI: Percutaneous coronary intervention; QALY: Quality-adjusted life-year.

**Table 8.** Scenario analysis 5 probabilistic results.

Scenario analysis 5 – Data from the Yuan *et al.* (2021) pragmatic minimum model used to estimate the probability of CA-AKI after PCI (5.24%) and the RR reduction in CA-AKI with DVA (7.06%) [17]		
Introduction of DVA		
	DVA	Current practice
Average cost per patient	£4340	£4650
QALYs per patient	9.065	9.040
Incremental costs per patient	-£310	
Incremental QALYs per patient	0.025	
Incremental cost–effectiveness ratio (costs/QALY)	Dominant	

CA-AKI: Contrast-associated acute kidney injury; DVA: Digital variance angiography; PCI: Percutaneous coronary intervention; QALY: Quality-adjusted life-year; RR: Relative risk.

## Discussion

This economic evaluation, exploring the cost–effectiveness of introducing DVA image processing alongside current practice during lower limb or abdominal endovascular procedures, compared with imaging using DSA alone, has demonstrated the potential cost–effectiveness of this innovative technology. Base-case results show a cost saving of £309 per patient over a lifetime horizon. QALY gains (+0.025) were also demonstrated, highlighting the clinical effectiveness of the intervention alongside its economic value.

As shown in one-way DSA, the cost saving potential of DVA image processing is heavily determined by its potential to reduce the risk of CA-AKI as well as costly cancerous events. In particular, the ability for DVA to reduce radiation exposure among patients undergoing imaging (70% reduction in exposure in the base-case analysis) is a strong determinant of cost savings given the high costs associated with lung and colorectal cancers. In our analysis, data from the York radiation model on the risk of cancer (per unit Gy) among males and females across age categories, as well as the probability of occurrence of each of the specific cancer types [46,47], were used in combination with data from Gyánó *et al.* on reduction in radiation exposure associated with DVA [1], to inform the overall reduction in the risk of each type of cancer when the intervention is used. The DSA shows that variation in the reduction in radiation exposure with DVA parameter shifts the final results by £99 per patient (-£360 when the higher value is used, and -£261 when the lower value is used), although the intervention remains cost saving in both cases.

Additionally, the one-way DSA exploring impact of parameter variation on incremental QALYs highlights that the most impactful parameters are the percentage reduction in the volume of CM used when DVA is available (50% in the base-case analysis), as well as the RR reduction associated with occurrence of CA-AKI when DVA is introduced alongside existing practice (8.23% in the base-case analysis). These results emphasize the importance of avoiding CA-AKI, as it relates to impact on patient quality-of-life. Robustness of these results has been validated in a Monte Carlo simulation analysis, with the cost–effectiveness of the intervention demonstrated in the base-case analysis. Notably, adding DVA to current practice imaging was found to be cost saving and more effective in 100% of the simulations performed, with the intervention also showing a 100% probability of being cost effective. Further scenario analyses exploring alternative parameter assumptions did not lead to a large deviation from the base-case results.

Previous work has been performed exploring the cost–effectiveness of image processing approaches, including DSA. A study by Hay *et al.* was comparable to our own analysis given its focus on the costs and outcomes of DSA compared with contrast-enhanced magnetic resonance angiography (CE-MRA) for imaging in lower extremity peripheral disease [52]. However, their findings that CE-MRA resulted in cost savings over DSA were based on a short-term analysis. The study of DVA presented here, therefore, considerably strengthens the economic evidence base in this clinical area given its focus on the recently developed DVA technique, as well as its consideration of lifetime costs and effects.

There are, however, limitations to the analysis which should be discussed. First, not all potential benefits associated with use of DVA could be captured in the economic model. For instance, improved visualization scores with DVA, which have been demonstrated in previous studies by Gyánó *et al.*, Thomas *et al.*, and Bastian *et al.* [1,9,12], are not considered directly. Thomas *et al.* reported that the overall visual scores with DVA were significantly higher than DSA scores among patients undergoing selective lower limb angiography [9]. However, for our economic analysis, insufficient data were available to inform the impact of improved visual score on subsequent patient treatment and long-term outcomes, including quality-of-life. As a result, this component was omitted from the analysis. Additionally, the model did not capture the potential opportunity for DVA image processing to be utilized among patients who otherwise would not be able to receive endovascular intervention, as part of a minimally invasive endovascular procedure, in other words, patients with estimated glomerular filtration rate (eGFR) 15 and/or severe iodine allergy. One may assume that had these additional benefits of DVA been included, the clinical and economic results for the intervention would have improved.

Secondly, the analysis was restricted to an NHS perspective, and therefore, the wider societal impact of DVA was not considered. While the consequences of a reduction in radiation exposure were assessed among patients in terms of long-term development of RI cancer, the impact on healthcare staff was not included in the analysis. As a result, the full benefits of DVA, beyond those directly impacting the patient population, are not factored into the economic analysis and it, therefore, does not fully reflect the potential cost savings of the technology. Therefore, the results may be regarded as a conservative estimate of cost savings, particularly when we take into account that the dose management values (50% contrast agent reduction; 70% radiation dose reduction) used in the base-case model will further improve due to the continuous development of DVA technology over time.

Finally, due to lack of alternative data, we used baseline patient characteristic information, as well as data on the association between reduction in CM volume and CA-AKI outcomes, based on information from Yuan *et al.* [17], which focused on a PCI population. However, according to expert clinical input, the assumption of comparable patient characteristics between patients undergoing lower limb, or abdomen, imaging and those who undergo PCI is clinically plausible. The association between CM volume and risk of CA-AKI may be independent of type of procedure performed, in which case the use of data based on an alternative patient population is justifiable. Alternative sources of data were explored in scenario analyses related to the change in probability of CA-AKI following reduction in CM volume, with the intervention remaining cost saving in all cases.

Despite the limitations presented above, a comprehensive economic model has been developed to represent the clinical pathways and health states that patients undergoing lower limb, or abdomen, x-ray angiography may follow. The long-term kidney- and cardiovascular-related complications, as well as the development of cancer, related to the two image processing methods being assessed, have been captured. This model is the first to assess the cost–effectiveness of introducing DVA alongside existing imaging practice, compared with use of DSA alone, and offers value to decision makers when selecting the optimal strategy in an English healthcare setting. In order to improve our comprehension of the relationship between CM volume and CA-AKI in lower limb imaging, it is necessary for future studies to conduct targeted investigations, and compare patient characteristics across various imaging procedures. These efforts will provide valuable insights to guide clinical practice in the context of lower limb imaging given that 385 patients underwent 390 lower limb x-ray angiography episodes between April 2022 and March 2023 in the UK [53].

## Conclusion

Introduction of the DVA image processing method alongside current practice improves imaging procedure safety and is a potentially cost-effective alternative to current practice imaging using DSA alone in lower-limb arterial recanalization, based on an analysis performed from an English NHS perspective. Potential long-term cost savings have been demonstrated, together with improved clinical outcomes associated to reduced CM volume and radiation exposure administered to patients.

## Summary points

- Digital variance angiography (DVA) has shown promising results in image processing compared with digital subtraction angiography (DSA), among patients undergoing lower limb x-ray angiography.
- This innovative technology has been shown to improve visualization during angiography, while reducing radiation exposure, contrast media (CM) volume, and related clinical complications such as contrast-associated acute kidney injury (CA-AKI) and radiation-induced (RI) cancers.
- This study aimed to explore the potential cost–effectiveness of using DVA alongside existing imaging practice, from an English NHS perspective.
- An economic model has been developed consisting of a decision tree and a Markov model, in order to respectively evaluate the short- and long-term costs and clinical outcomes associated with introduction of DVA based on the resulting reduction in CM and radiation exposure.
- DVA has been shown to result in an average cost saving of £309 per patient over a lifetime horizon.
- A quality-adjusted life-year gain of 0.025 per patient with DVA, has been demonstrated over the model duration.
- Key model parameters include the occurrence of costly cancerous events, including lung and colorectal cancer, which DVA has been shown to reduce, as well as the reduction in the risk of developing CA-AKI.
- The clinical and economic benefits offered by DVA suggest that this technology should be considered as an alternative to existing image processing methods.

## Supplementary Material



## References

[1] Gyánó M, Berczeli M, Csobay-Novák C Digital variance angiography allows about 70% decrease of DSA-related radiation exposure in lower limb X-ray angiography. Sci. Rep. 11, 21790 (2021). 34750427 10.1038/s41598-021-01208-3 PMC8575921

[2] Sótonyi P, Berczeli M, Gyánó M Radiation exposure reduction by digital variance angiography in lower limb angiography: a randomized controlled trial. J. Cardiovasc. Dev. Dis. 10, 198 (2023). 37233165 10.3390/jcdd10050198 PMC10219428

[3] Okamoto K, Ito J, Sakai K, Yoshimura S. The principle of digital subtraction angiography and radiological protection. Interv. Neuroradiol. 6(Suppl. 1), 25–31 (2000). 20667218 10.1177/15910199000060S102 PMC3685929

[4] Rogers K. Angiography | Medicine. Encyclopedia Britannica (2022). (Accessed 23 June 2022). https://www.britannica.com/science/angiography#ref293758

[5] Jeans WD. The development and use of digital subtraction angiography. Br. J. Radiol. 63(747), 161–168 (1990). 2185864 10.1259/0007-1285-63-747-161

[6] Jalali A, Srinivasan VM, Chinnadurai P, Kan P, Arthur A, Duckworth EA. Two-color 3D-3D fusion of selective rotational cerebral angiograms: a novel approach to imaging in cerebrovascular neurosurgery. J. Neurointerv. Surg. 8(10), 1056–1060 (2016). 26574481 10.1136/neurintsurg-2015-011963

[7] Kato N, Yuki I, Hataoka S 4D digital subtraction angiography for the temporal flow visualization of intracranial aneurysms and vascular malformations. J. Stroke Cerebrovasc. Dis. 29(12), 105327 (2020). 32992207 10.1016/j.jstrokecerebrovasdis.2020.105327

[8] Szigeti K, Máthé D, Osváth S. Motion based x-ray imaging modality. IEEE Trans. Med. Imaging 33(10), 2031–2038 (2014). 24951684 10.1109/TMI.2014.2329794

[9] Thomas RP, Bastian MB, Viniol S Digital variance angiography in selective lower limb interventions. J. Vasc. Interv. Radiol. 33(2), 104–112 (2022). 34653607 10.1016/j.jvir.2021.09.024 PMC8844582

[10] Gyánó M, Góg I, Óriás VI Kinetic imaging in lower extremity arteriography: comparison to digital subtraction angiography. Radiology 290(1), 246–253 (2019). 30325284 10.1148/radiol.2018172927

[11] Óriás VI, Gyánó M, Góg I Digital variance angiography as a paradigm shift in carbon dioxide angiography. Invest. Radiol. 54(7), 428–436 (2019). 30829769 10.1097/RLI.0000000000000555

[12] Bastian MB, König AM, Viniol S Digital variance angiography in lower-limb angiography with metal implants Cardiovasc. Intervent. Radiol. 44, 452–459 (2021). 10.1007/s00270-020-02697-x PMC7864852 33145701

[13] Weinstein MC, O'Brien B, Hornberger J , ISPOR Task Force on Good Research Practices--Modeling Studies. Principles of good practice for decision analytic modeling in health-care evaluation: report of the ISPOR Task Force on Good Research Practices--Modeling Studies. Value Health 6(1), 9–17 (2003). 12535234 10.1046/j.1524-4733.2003.00234.x

[14] NICE. NICE Health Technology Evaluations: The Manual (2023). https://www.nice.org.uk/process/pmg36/resources/nice-health-technology-evaluations-the-manual-pdf-72286779244741

[15] Mandurino-Mirizzi A, Munafò A, Crimi G. Contrast-associated acute kidney injury. J. Clin. Med. 11(8), 2167 (2022). 35456260 10.3390/jcm11082167 PMC9027950

[16] Ons.gov.uk. National life tables: England - Office for National Statistics. (2022). (Accessed 23 June 2022). https://www.ons.gov.uk/peoplepopulationandcommunity/birthsdeathsandmarriages/lifeexpectancies/datasets/nationallifetablesenglandreferencetables/current

[17] Yuan N, Latif K, Botting PG Refining safe contrast limits for preventing acute kidney injury after percutaneous coronary intervention. J. Am. Heart Assoc. 10(1), e018890 (2021). 33325246 10.1161/JAHA.120.018890 PMC7955500

[18] Dangas G, Iakovou I, Nikolsky E Contrast-induced nephropathy after percutaneous coronary interventions in relation to chronic kidney disease and hemodynamic variables. Am. J. Cardiol. 95(1), 13–19 (2005). 15619387 10.1016/j.amjcard.2004.08.056

[19] Mehran R, Aymong ED, Nikolsky E A simple risk score for prediction of contrast-induced nephropathy after percutaneous coronary intervention: development and initial validation. J. Am. Coll. Cardiol. 44(7), 1393–1399 (2004). 15464318 10.1016/j.jacc.2004.06.068

[20] Mueller C, Buerkle G, Buettner HJ Prevention of contrast media-associated nephropathy: randomized comparison of 2 hydration regimens in 1620 patients undergoing coronary angioplasty. Arch. Intern. Med. 162(3), 329–336 (2002). 11822926 10.1001/archinte.162.3.329

[21] Óriás VI, Szöllősi D, Gyánó M Initial evidence of a 50% reduction of contrast media using digital variance angiography in endovascular carotid interventions. Eur. J. Radiol. Open 7, 100288 (2020). 33294499 10.1016/j.ejro.2020.100288 PMC7683322

[22] Juszkat R, Blaszak MA, Majewska N, Majewski W. Dose-area product of patients undergoing digital subtraction angiography (DSA), abdominal aorta and lower limb examinations. Health Phys. 96(1), 13–18 (2009). 19066482 10.1097/01.HP.0000326445.75429.a2

[23] Feldman DA, Shroff AR, Bao H, Curtis JP, Minges KE, Ardati AK. Stent selection among patients with chronic kidney disease: results from the NCDR CathPCI Registry. Catheter Cardiovasc Interv. 96(6), 1213–1221 (2020). 31909543 10.1002/ccd.28698

[24] James MT, Hemmelgarn BR, Wiebe N Glomerular filtration rate, proteinuria, and the incidence and consequences of acute kidney injury: a cohort study. Lancet 376(9758), 2096–2103 (2010). 21094997 10.1016/S0140-6736(10)61271-8

[25] Eriksen BO, Ingebretsen OC. The progression of chronic kidney disease: a 10-year population-based study of the effects of gender and age. Kidney Int. 69(2), 375–382 (2006). 16408129 10.1038/sj.ki.5000058

[26] NICE. Clinical Guideline 169. Acute kidney injury: prevention, detection and management [CG169]. National Institute for Health and Clinical Excellence, Royston, UK (2013).

[27] Coca SG, Singanamala S, Parikh CR. Chronic kidney disease after acute kidney injury: a systematic review and meta-analysis. Kidney Int. 81(5), 442–448 (2012). 22113526 10.1038/ki.2011.379 PMC3788581

[28] Valle JA, McCoy LA, Maddox TM Longitudinal risk of adverse events in patients with acute kidney injury after percutaneous coronary intervention: insights from the National Cardiovascular Data Registry. Circ. Cardiovasc. Interv. 10(4), e004439 (2017). 28404621 10.1161/CIRCINTERVENTIONS.116.004439

[29] Smolina K, Wright FL, Rayner M, Goldacre MJ. Long-term survival and recurrence after acute myocardial infarction in England, 2004 to 2010. Circ. Cardiovasc. Qual. Outcomes 5(4), 532–540 (2012). 22740013 10.1161/CIRCOUTCOMES.111.964700

[30] NICE. Medical Technologies Guidance 60. DyeVert Systems for reducing the risk of acute kidney injury in coronary and peripheral angiography [MTG60]. National Institute for Health and Clinical Excellence, UK (2021).

[31] Villar E, Remontet L, Labeeuw M, Ecochard R. Effect of age, gender, and diabetes on excess death in end-stage renal failure. J. Am. Soc. Nephrol. 18(7), 2125 (2007). 17582163 10.1681/ASN.2006091048

[32] NICE. Technology Appraisal Guidance 236. Ticagrelor for the treatment of acute coronary syndromes [TA236]. National Institute for Health and Clinical Excellence, UK (2011).

[33] Jesky MD, Dutton M, Dasgupta I Health-related quality of life impacts mortality but not progression to end-stage renal disease in pre-dialysis chronic kidney disease: a prospective observational study. PLOS ONE 11(11), e0165675 (2016). 27832126 10.1371/journal.pone.0165675 PMC5104414

[34] Lee AJ, Morgan CL, Conway P, Currie CJ. Characterisation and comparison of health-related quality of life for patients with renal failure. Curr. Med. Res. Opin. 21(11), 1777–1783 (2005). 16307698 10.1185/030079905X65277

[35] Cooper JT, Lloyd A, Sanchez JJG, Sörstadius E, Briggs A, McFarlane P. Health related quality of life utility weights for economic evaluation through different stages of chronic kidney disease: a systematic literature review. Health Qual. Life Outcomes 18(1), 310 (2020). 32957990 10.1186/s12955-020-01559-x PMC7507735

[36] Sullivan PW, Slejko JF, Sculpher MJ, Ghushchyan V. Catalogue of EQ-5D scores for the United Kingdom. Med. Decis. Making 31(6), 800–804 (2011). 21422468 10.1177/0272989X11401031

[37] NICE Technology Assessment Report. Diagnostics Assessment Report – EOS 2D/3D X-ray Imaging System, Final Report (16^th^ March 2011).

[38] Department of Health. NHS Reference Costs 2019/20. Collection Guidance. Department of Health, UK (2020).

[39] Walker S, Asaria M, Manca A Long-term healthcare use and costs in patients with stable coronary artery disease: a population-based cohort using linked health records (CALIBER). Eur. Heart J. Qual. Care Clin. Outcomes 2(2), 125–140 (2016). 27042338 10.1093/ehjqcco/qcw003 PMC4816202

[40] Subramanian S, Tumlin J, Bapat B, Zyczynski T. Economic burden of contrast-induced nephropathy: implications for prevention strategies. J. Med. Econ. 10(2), 119–134 (2007). 19702434 10.3111/200710119134

[41] Kerr M, Bedford M, Matthews B, O'Donoghue D. The economic impact of acute kidney injury in England. Nephrol. Dial. Transplant. 29(7), 1362–1368 (2014). 24753459 10.1093/ndt/gfu016

[42] Tripathi B, Sharma P, Arora S Safety and feasibility of robotic assisted percutaneous coronary intervention compared to standard percutaneous coronary intervention – a systematic review and meta-analysis. Indian Heart J. 73(5), 549–554 (2021). 34627567 10.1016/j.ihj.2021.08.006 PMC8514414

[43] King's Health Partners and Royal Brompton & Harefield NHS Foundation Trust. Feasibility Study (2018). (Accessed 23 June 2022). https://www.rbht.nhs.uk/sites/nhs/files/Governors/Other%20papers/King%E2%80%99s%20Health%20Partners%20and%20Royal%20Brompton%20%26%20Harefield%20NHS%20Foundation%20Trust%20Feasibility%20Study%20APPENDIX%20FINAL%20DRAFT%20VERSION%2020%20April%202018.pdf

[44] Work-day.co.uk. Business days calculator in the UK and Ireland. (2022). (Accessed 23 June 2022). https://www.work-day.co.uk/

[45] Fletcher D, Edwards D, Tolchard S Improving theatre turnaround time. BMJ Open Quality 6(1), u219831.w8131 (2017). 10.1136/bmjquality.u219831.w8131 PMC5306684 28243441

[46] Westwood M, Al M, Burgers L A systematic review and economic evaluation of new-generation computed tomography scanners for imaging in coronary artery disease and congenital heart disease: Somatom Definition Flash, Aquilion ONE, Brilliance iCT and Discovery CT750 HD. Health Technol. Assess. 17(9), 1–243 (2013). 10.3310/hta17090 PMC4781122 23463937

[47] McKenna C, Wade R, Faria R EOS 2D/3D X-ray imaging system: a systematic review and economic evaluation. Health Technol. Assess. 16(14), 1–188 (2012). 10.3310/hta16140 PMC4781036 22449757

[48] National Research Council (US) Committee on the Biological Effects of Ionizing Radiation (BEIR V). Health Effects of Exposure to Low Levels of Ionizing Radiation: Beir V. National Academies Press, WA, USA (1990). 25032334

[49] Joint Formulary Committee. British National Formulary 83. BMJ Publishing and the Royal Pharmaceutical Society, UK (2020).

[50] NICE. Clinical Guideline 169. Acute Kidney Injury: Prevention, Detection and Management (CG169). National Institute for Health and Clinical Excellence, UK (2013).

[51] Gurm HS, Seth M, Mehran R Blue Cross Blue Shield of Michigan Cardiovascular Consortium (BMC2). Impact of contrast dose reduction on incidence of acute kidney injury (AKI) among patients undergoing PCI: a modeling study. J. Invasive Cardiol. 28(4), 142–146 (2016). 26773238

[52] Hay JW, Lawler E, Yucel K Cost impact of diagnostic imaging for lower extremity peripheral vascular occlusive disease. Value Health 12(2), 262–266 (2009). 18657093 10.1111/j.1524-4733.2008.00438.x

[53] NHS Digital. National Health Service Data Set (2023). https://www.nhsdigital.nhs.uk/data

