# Supplementary material for: Digital variance angiography in patients undergoing lower limb arterial recanalization: cost–effectiveness analysis within the English healthcare setting
Source: J Comp Eff Res. 2024 Mar 22;13(4):e230068. doi: 10.57264/cer-2023-0068 (PMC11044957; doi:10.57264/cer-2023-0068)
Supplement: Supplementary file 1 [file cer-13-230068-s1.docx]

Supplementary Materials

When data from Yuan et al. 2021 [15] were used, the probability of CA-AKI following PCI with current practice was estimated based on the safe CM volume limit. The following formula was used to estimate this value:

$AKI \left( patient characteristics,safe contrast media volume \right)= 1/(1+exp(-(a*contrast media volume without DVA+sumproduct(coefficients of risk factors for CA-AKI, corresponding patient characteristics)+b)))$,

where a and b are constant values, as derived from Yuan et al. 2021. Different associated values for the risk factors could be used to estimate the alternative values for the full, pragmatic full and pragmatic minimum models [15].
